# Assessing Cofactor Usage in *Pseudoclostridium thermosuccinogenes* via Heterologous Expression of Central Metabolic Enzymes

**DOI:** 10.3389/fmicb.2019.01162

**Published:** 2019-05-24

**Authors:** Jeroen Girwar Koendjbiharie, Kimberly Wevers, Richard van Kranenburg

**Affiliations:** ^1^Corbion, Gorinchem, Netherlands; ^2^Laboratory of Microbiology, Wageningen University & Research, Wageningen, Netherlands

**Keywords:** *Pseudoclostridium thermosuccinogenes*, cofactor specificity, GTP, pyrophosphate, 6-phosphofructokinase, glyceraldehyde 3-phosphate dehydrogenase, energy charge

## Abstract

*Pseudoclostridium thermosuccinogenes* and *Hungateiclostridium thermocellum* are being studied for their potential to contribute to a more sustainable bio-based economy. Both species were shown previously to rely on GTP or pyrophosphate instead of ATP as cofactors in specific reactions of central energy metabolism for reasons that are not well understood yet. Since it is often impossible to predict cofactor specificity from the primary protein structure, thirteen enzymes from *P. thermosuccinogenes* were cloned and heterologous expressed in *Escherichia coli* to assess the cofactor usage *in vitro* and paint a more complete picture of the cofactor usage in the central metabolism of *P. thermosuccinogenes*. The assays were conducted with heat-treated *E. coli* cell-free extract devoid of background activity to allow the quick assessment of a relatively large number of (thermophilic) enzymes. Selected enzymes were also purified to allow the determination of the enzyme kinetics for competing cofactors. Following the results of the glucokinase (GK), galactokinase, xylulokinase (XK), and ribokinase assays, it seems that phosphorylation of monosaccharides by and large is mainly GTP-dependent. Some possible implications of this relating to the adenylate/guanylate energy charge are discussed here. Besides the highly expressed pyrophosphate-dependent 6-phosphofructokinase, another 6-phosphofructokinase was found to be equally dependent on ATP and GTP, while no 6-phosphofructokinase activity could be demonstrated for a third. Both type I glyceraldehyde 3-phosphate dehydrogenases were found to be NAD^+^-dependent, and further, acetate kinase, isocitrate dehydrogenase, and three enzymes predicted to be responsible for the interconversion of phosphoenolpyruvate and pyruvate (i.e., pyruvate kinase; pyruvate, phosphate dikinase; phosphoenolpyruvate synthase), were also assessed.

## Introduction

*Pseudoclostridium thermosuccinogenes* and *Hungateiclostridium thermocellum*, thermophilic bacteria belonging to the *Hungateiclostridiaceae* have been shown to rely on GTP and pyrophosphate (PP_i_), as well as the “typical” ATP in their central metabolism (Zhou et al., 2013; Koendjbiharie et al., 2018). Both are being studied for their potential to contribute to the transition toward a more sustainable bio-based economy. *H. thermocellum* for its unrivaled capability to degrade cellulosic biomass (Kothari et al., 2018), and *P. thermosuccinogenes* because it is the only known thermophile to produce succinic acid, a precursor for bioplastics, as one of the main products of fermentation (Drent et al., 1991; Sridhar and Eiteman, 2001; Koendjbiharie et al., 2018). The use of thermophiles in industry has the advantage that less energy is required for cooling the large reactors they are grown in, and that simultaneous saccharification and fermentation is possible when the optimal temperature of the commercial fungal cellulases overlaps with the growth temperature of the fermenting microorganism (Olofsson et al., 2008; Ou et al., 2009). Nevertheless, for either organism to be used successfully in any industrial process, it is crucial to have an in-depth understanding of their physiology. In particular the characteristics of their complex central energy metabolism should be unraveled, as significant metabolic engineering will be required to improve production yields, rates, and titres (Nielsen and Keasling, 2016).

One aspect that is still not well understood is why PP_i_ and GTP are used in addition to ATP as phosphoryl carriers in the central metabolism, rather than just ATP. PP_i_ is used for the conversion of fructose 6-phosphate to fructose 1,6-bisphosphate by 6-phosphofructokinase (6-PFK) and for the conversion of phosphoenolpyruvate (PEP) to pyruvate by pyruvate, phosphate dikinase (PPdK). By utilizing PP_i_, a “waste” product of many anabolic reactions that would otherwise simply be hydrolysed, ATP-equivalents are being conserved (Mertens, 1991). Analogous to that, using PP_i_ allows PPdK to form ATP from AMP, instead of ADP as is done by pyruvate kinase (PK). However, it was already calculated that PP_i_ formation from anabolism by no means accounts for the total PP_i_ requirement for 6-PFK alone, suggesting that an additional PP_i_ production mechanism exists (Zhou et al., 2013). Possible mechanisms include a proton pumping pyrophosphatase, or a cycle involving the simultaneous formation and degradation of glycogen. In such a cycle, glucose 1-phosphate and ATP are converted to ADP-glucose and PP_i_. ADP-glucose is used to generate glycogen, releasing the ADP, after which glucose 1-phosphate is regenerated from glycogen combined with orthophosphate, leading to the net formation of ADP and PP_i_ from ATP and orthophosphate. Much more unclear even is the use of GTP as alternative to ATP. Both *P. thermosuccinogenes* and *H. thermocellum* have a GTP-dependent glucokinase (GK) and PEP carboxykinase (PEPCK), and *P. thermosuccinogenes* also has a GTP-dependent xylulokinase (XK) (Zhou et al., 2013; Koendjbiharie et al., 2018). It was speculated to represent a “simple” regulatory mechanism, via the direct link to protein synthesis, or that a guanylate energy charge could exist that is different from the adenylate energy charge, allowing GTP and ATP to fulfill different roles in the metabolism, analogous to NADH and NADPH having different oxidation states in the cell (Koendjbiharie et al., 2018). The adenylate energy charge is defined as [(ATP) + ½12 (ADP)]/[(ATP) + (ADP) + (AMP)] (Atkinson, 1968).

Furthermore, the finding that GK and XK in these organisms rely on GTP instead of ATP also highlights the fact that it is very often impossible to confer cofactor usage from the amino acid sequence alone. Either because too few (closely related) enzymes of the kind have been experimentally characterized (for cofactor usage) to find a certain consensus sequence or a correlation to phylogenetic groups, or because it is simply not possible to deduce it from the sequence. For this reason it is valuable to conduct basic characterization of metabolic enzymes, in particular from non-model organisms, in order to learn more about cofactor usage.

In this study, we cloned and expressed thirteen genes of the central metabolism of *P. thermosuccinogenes* into *Escherichia coli*. Cell-free extracts (CFE) of *E. coli* expressing these enzymes were then used to assess the enzymes’ activities and cofactor specificities. Selected enzymes were purified to assess the kinetics in more detail. Furthermore, hypotheses for the use of GTP and PP_i_ next to ATP are formulated.

## Materials and Methods

### Cloning of *P. thermosuccinogenes* Genes in *E. coli*

The primers used to clone the thirteen *P. thermosuccinogenes* genes that were characterized in this study are listed in Table 1. The genes were cloned into a pET-28b(+) (Novagen, Madison, WI, United States) derived backbone, generated using primers ATTGGATTGGAAGTACAGGTTTTCATGGTGATGGTGATGGTGAGAAGAACCCATGGTATATCTCCTTCTTAAAG and ATTGGAAGTGGATAACGGATCCGAATTCGAGCGCCGTCGACAAGCTTGCGG, as described previously (Koendjbiharie et al., 2018). In the final constructs, the cloned enzymes have an N-terminal His_6_-tag flanking a TEV protease site. The constructs were initially transformed to *E. coli* DH5α. After verification via pyrosequencing (Macrogen), the plasmids were transformed to *E. coli* Rosetta (Novagen), an *E. coli* BL21 derivative containing the pRARE plasmid encoding tRNAs of rare codons in *E. coli* and a chloramphenicol resistance marker.

**Table 1:** Primers used to clone the selected *P. thermosuccinogenes* genes into pET-28b(+).

Locus tag	Primers
CDQ83_02810	TACTTCCAATCCAATGCAGATATTAATCAATTAAAGCAAAAAT
	TCATT
	TTATCCACTTCCAATGTTACTTAATCTCCCTGCCT
CDQ83_03295	TACTTCCAATCCAATGCAATGGAGGGTCAAGTAAAAATAC
	TTATCCACTTCCAATGCTATCCTTCAAGCCCC
CDQ83_03625	TACTTCCAATCCAATGCAGAAATTTACGAAAAGGTTAGC
	TTATCCACTTCCAATGCTATTTACATATGAGCTTTTGG
CDQ83_04880	TACTTCCAATCCAATGCAAGTACGAAAGTTGGAATTAAC
	TTATCCACTTCCAATGTCATATGCTTGAAGATACATATG
CDQ83_07070	TACTTCCAATCCAATGCAGTAAAGGTTGGAGTGGC
	TTATCCACTTCCAATGTTACTTCCATTTTCCCATCC
CDQ83_07225	TACTTCCAATCCAATGCACCTGATATAAGAACTATAGGAGTC
	TTATCCACTTCCAATGTTATAAGGCCAGTATCCTG
CDQ83_07295	TACTTCCAATCCAATGCAAAAGTTTTGGTTATCAATGC
	TTATCCACTTCCAATGTTATTTGCTCAATATAGCCACT
CDQ83_07455	TACTTCCAATCCAATGCAACAAAGTATGTTTATCTTTTTAG
	TGAAG
	TTATCCACTTCCAATGTTATTTATTTTTAATGGCAGCTTGAG
CDQ83_08625	TACTTCCAATCCAATGCAGAAAAAATCAAAATGCGAGTTC
	TTATCCACTTCCAATGTCAAAGGGTTTGCTCC
CDQ83_09600	TACTTCCAATCCAATGCAAGAAAAACAAAAATAATCTGTACAT
	TTATCCACTTCCAATGTTAGTTCTCAGCGTCTG
CDQ83_10590	TACTTCCAATCCAATGCAGCAGTAAAGATAGGTATTAATGG
	TTATCCACTTCCAATGTTATTTAGCGTCAACTTCAG
CDQ83_10650	TACTTCCAATCCAATGCAAAGAAACGTATTGGAGTGTT
	TTATCCACTTCCAATGTTAATCCCCAAAACTTACCC
CDQ83_11320	TACTTCCAATCCAATGCAGCTGAATTAAAAGGCGC
	TTATCCACTTCCAATGTTATTTAGTTGCCAATACTTTCTTAAG

### Preparation of Cell-Free Extracts (CFE)

Rosetta strains carrying the expression plasmids, including an empty vector control, were grown overnight in 5 ml LB containing 50 μg/ml kanamycin and 20 μg/ml chloramphenicol at 37°C. The next day, overnight cultures were added to 50 ml pre-warmed LB with antibiotics, and grown to an OD_600_ of 0.6 to 0.8 at 37°C, after which they were placed on ice for 20 min. Heterologous gene expression was then induced by the addition of 0.2 mM IPTG (isopropyl-β-D-thiogalactopyranoside). Following an additional incubation step of 3 to 4 h at 37°C, cells were harvested via centrifugation at 4,800 × *g* for 10 min at 4°C, and were washed twice with cold 50 mM MOPS buffer (pH 7.0 at room temperature). Cells were resuspended in 5 ml MOPS buffer containing cOmplete^TM^, mini, EDTA-free protease inhibitor cocktail, 1 tablet per ∼10 mL (Roche). Cells were lysed using a French press at ∼120 kPa. Lysate was centrifuged at 20.000 × *g* for 10 min at 4°C. The supernatant (i.e., the non-heated CFE) was split in two fractions, of which one was incubated at 60°C for 30 min. Precipitated proteins were removed by another centrifugation step, leaving the heat-treated CFE. The Bradford assay was used to measure the total protein concentration in the heated and non-heated extracts, which were also run on a SDS-PAGE gel to verify that heterologous expression was successful. Pictures of the gels can be found in the Supplementary Figures S1–S3.

### Enzyme Purification

For affinity chromatography of selected enzymes, lysates were generated in identical fashion as for the CFEs, except that larger cultures were used (0.5 l LB for ribokinase and galactokinase, and 2 l for ATP/GTP-dependent phosphofructokinase), and that the wash/resuspension buffer contained 50 MOPS buffer (pH 7.4 at room temperature) with 20 mM imidazole. A HisTrap^TM^ HP column (GE Healthcare; optimal at pH 7.4) with an ÄKTA pure FPLC system were used for the purification. Elution was done over a gradient with the same buffer containing 500 mM imidazole. The buffer of the eluted protein was then exchanged with 50 mM MOPS (pH 7.0 at room temperature) using an Amicon^^®^^ ultra centrifugal filter (Merck) with a nominal molecular weight limit of 10.000 Da. SDS-PAGE was used to verify purity.

### Enzyme Assays

The enzyme assays in this study are all based on the measurement of NAD(P)H at 340 nm (ε = 6.2 mM^−1^cm^−1^), which is produced or consumed either directly by the investigated enzyme, or via the coupling to one or more auxiliary enzymes. A Shimadzu U-2010 spectrophotometer in combination with a thermoelectric cell holder was used for the measurements, which were performed at 55°C. Quartz cuvettes containing 1 ml of the reaction mixture were used with a 1.0-cm path length. Activities are expressed in micromoles of product per minute per mg of CFE protein. The enzymes and biochemicals were obtained from Sigma. 5 to 100 μl CFE was used, that in some cases had to be diluted first. At least three different concentrations of CFE were used for each assay, to verify that the enzymes in the extract were the limiting factor, and not the auxiliary enzymes.

**FIGURE 1:**
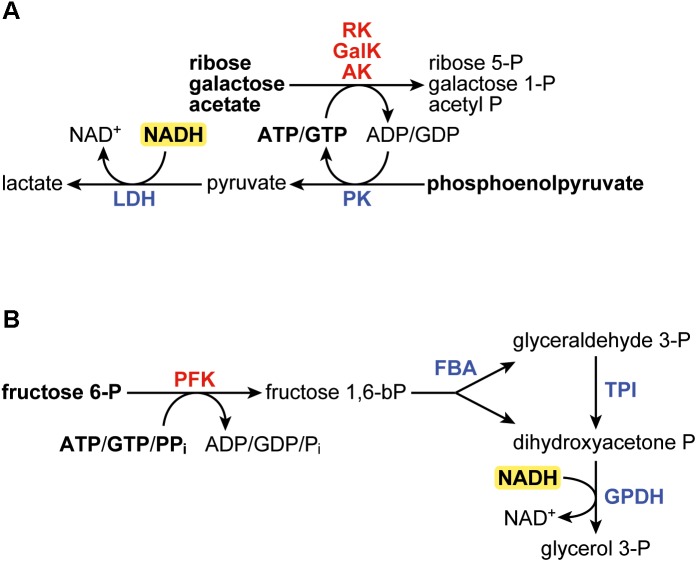
**(A)** Scheme of the coupled reactions in the assays for ribokinase (RK), galactokinase (GalK), and acetate kinase (AK). **(B)** Scheme of the coupled reactions in the 6-phosphofructokinase assay. In bold are the components added in to the reaction. LDH, lactate dehydrogenase; PK, pyruvate kinase; FBA, fructose bisphosphate aldolase; TPI, triose phosphate isomerase; GPDH, glycerol-3-phosphate dehydrogenase.

The ribokinase (EC 2.7.1.15) assay was based on the XK assay described elsewhere (Dills et al., 1994; Koendjbiharie et al., 2018) and contained 50 mM MOPS (pH 7.0 at room temperature), 10 mM MgCl_2_, 100 mM KCl, 5 mM D-ribose, 2 mM ATP, or GTP, 0.15 mM NADH, 2 mM phosphoenolpyruvate, 4 U/ml PK (rabbit), 4 U/ml lactate dehydrogenase (rabbit). Ribose was added to start the reaction. Since the stock solutions of ATP and in particular GTP contain ADP/GDP impurities, ribose was added only after the OD_340_ had stabilized. The galactokinase (EC 2.7.1.6) and acetate kinase (EC 2.7.2.1) were conducted identically to the ribokinase assay, except that 5 mM D-galactose and 5 mM potassium acetate, respectively, were used instead of D-ribose. Figure 1A shows a graphical scheme of the coupled reactions in the three assays. In the kinetics assays with purified enzymes, ATP or GTP were added at varied concentrations. The Michaelis–Menten equation was fitted to the data by minimizing the sum of the squares of the vertical differences, in order to find K_M_ and k_cat_. The data and the fitted model can be found in the Supplementary Material.

The 6-phosphofructokinase (EC 2.7.1.11; EC 2.7.1.90) assay was adapted from Zhou et al. (2013) and contained 50 mM MOPS (pH 7.0 at room temperature), 5 mM MgCl_2_, 1 mM fructose 6-phosphate, 2 mM ATP, GTP, or pyrophosphate, 0.15 mM NADH, 4 U/ml aldolase (rabbit), 4 U/ml triosephosphate isomerase (rabbit), and 4 U/ml glycerol-3-phosphate dehydrogenase (rabbit). Fructose 6-phosphate was added to start the reaction. Figure 1B shows a graphical scheme of the coupled assays. Since two equivalents of NADH are oxidized per equivalent of fructose 6-phosphate that is phosphorylated, the final rates were adjusted for this. In the kinetics assays with purified enzymes, ATP or GTP were added to start the reaction, at varied concentrations. The Michaelis–Menten equation was fitted to the data by minimizing the sum of the squares of the vertical differences, in order to find K_M_ and k_cat_. The data and the fitted model can be found in the Supplementary Material.

The pyruvate kinase (EC 2.7.1.40) assay was adapted from Zhou et al. (2013) and contained 50 mM MOPS (pH 7.0 at room temperature), 10 mM MgCl_2_, 100 mM KCl, 2 mM phosphoenolpyruvate, 2 mM ADP, or GDP, 0.15 mM NADH, and 4 U/ml lactate dehydrogenase (rabbit). Phosphoenolpyruvate was added to start the reaction.

The pyruvate, phosphate dikinase (EC 2.7.9.1) assay was adapted from Reeves (1968) and contained 50 mM MOPS (pH 7.0 at room temperature), 5 mM MgCl_2_, 10 mM KCl, 20 mM NH_4_Cl, 2 mM phosphoenolpyruvate, 2 mM AMP, or GMP, 2 mM pyrophosphate, 0.15 mM NADH, and 4 U/ml lactate dehydrogenase (rabbit). Phosphoenolpyruvate was added to start the reaction.

The phosphoenolpyruvate synthase (EC 2.7.9.2) assay was adapted from Imanaka et al. (2006) and contained 50 mM MOPS (pH 7.0 at room temperature), 5 mM MgCl_2_, 10 mM KCl, 20 mM NH_4_Cl, 2 mM phosphoenolpyruvate, 2 mM AMP, or GMP, 5 mM K_2_HPO_4_, 0.15 mM NADH, and 4 U/ml lactate dehydrogenase (rabbit). Phosphoenolpyruvate was added to start the reaction.

The glyceraldehyde 3-phosphate dehydrogenase (EC 1.2.1.12) assay was adapted from Byers (1982) and contained 50 mM Tris (pH 8.5 at 60°C), 5 mM MgCl_2_, 4 mM dihydroxyacetone phosphate, 0.15 mM NAD^+^, or NADP^+^, 5 mM sodium arsenate, and 4 U/ml triosephosphate isomerase (rabbit). Dihydroxyacetone phosphate was added to start the reaction. For our convenience, dihydroxyacetone phosphate, and triosephosphate isomerase were used instead of glyceraldehyde 3-phosphate directly. For glyceraldehyde 3-phosphate dehydrogenase assays sodium arsenate is typically used instead of orthophosphate, as the incorporated arsenate is spontaneously hydrolyzed, driving the reaction forward. The elevated pH of 8.5 is also crucial to shift the thermodynamic equilibrium toward 1,3-bisphosphoglycerate-forming direction.

The isocitrate dehydrogenase (EC 1.1.1.42) assay was adapted from Plaut (1962) and contained 50 mM MOPS (pH 7.0 at room temperature), 5 mM MgCl_2_, 100 mM NaCl, 0.75 mM NAD^+^, or NADP^+^, and 5 mM isocitrate, which was added to start the reaction.

To test ribokinase and galactokinase activity with pyrophosphate, Malachite Green Phosphate Assay Kit (Sigma) was used to detect to formation of orthophosphate by purified enzymes. The reaction contained 50 mM MOPS (pH 7.0 at room temperature), 1 mM MgCl_2_, 50 mM KCl, 5 mM D-ribose/galactose, and 2 mM pyrophosphate. Reaction was conducted at 55°C. Samples were taken every 2 min, and placed on ice until analysis.

## Results

Thirteen genes from *P. thermosuccinogenes* involved in the central carbon metabolism were heterologously expressed in *E. coli* in order to investigate the cofactor usage. Most of those were involved in phosphoryl transfer (Tables 2, 3). The three 6-PFK orthologs were tested for ATP, GTP, and PP_i_. Ribokinase (RK), galactokinase (GalK), and acetate kinase (AK) were only tested for ATP and GTP, since their assays relied on the coupling with auxiliary PK and lactate dehydrogenase using PEP, to detect NDP formation, which would not work in the case PP_i_ was used. The assays were carried out using the CFEs from *E. coli*, without any additional purification (e.g., affinity chromatography), except for an *E. coli* protein-denaturing heating step. We previously showed that this method was a quick and simple method for basic characterization of enzymes (Koendjbiharie et al., 2018). For all assays, CFE with an empty vector was used as a control, and in all cases no significant background activity was present.

**Table 2:** Enzyme assays with *E. coli* cell-free extract containing several heterologously expressed enzymes from *P. thermosuccinogenes*, to determine the cofactors involved in phosphoryl transfer (ATP, GTP, or PP_i_).

Assay	Locus tag	Annotation	Activity		
			ATP	GTP	PP_i_
Ribokinase	CDQ83_03295	ribokinase	36 ± 3	54 ± 2	–
Galactokinase	CDQ83_02810	galactokinase	5.1 ± 0.5	33 ± 1	–
Acetate Kinase^∗∗^	CDQ83_07295	acetate kinase	71 ± 7	10 ± 0	–
6-Phosphofructokinase	CDQ83_07225	6-phosphofructokinase	13 ± 1	13 ± 5	ND^∗^
	CDQ83_10650	6-phosphofructokinase	ND	ND	ND
	CDQ83_11320	6-phosphofructokinase	ND	ND	220 ± 26

*Reaction velocities are given in μmol/mg cell-free extract protein/min ± standard deviation. ^∗^Not detected: value close to zero and not significantly different from empty vector control. ^∗∗^Non-heated extracts were used, due to the apparent low thermo-stability of the acetate kinase from P. thermosuccinogenes.*

**Table 3:** Enzyme assays with *E. coli* cell-free extract containing heterologously expressed enzymes from *P. thermosuccinogenes* involved in the direct conversion of phosphoenolpyruvate to pyruvate, i.e., pyruvate kinase; pyruvate, phosphate dikinase; and phosphoenolpyruvate synthase.

Locus tag	Annotation	Pyruvate kinase activity		PPdK activity		PEP synthase activity	
		ADP	GDP	AMP	GMP	AMP	GMP
CDQ83_03625	pyruvate kinase	ND^∗^	ND	ND	ND	ND	ND
CDQ83_07455	pyruvate, phosphate dikinase	ND	ND	11 ± 1	ND	ND	ND
CDQ83_09600	pyruvate kinase	52 ± 1	34 ± 1	ND	ND	ND	ND

*Their cofactor usage for those three reactions were assayed. Reaction velocities are given in μmol/mg cell extract protein/min ± standard deviation.^∗^Not detected: value close to zero and not significantly different from empty vector control.*

Ribokinase, responsible for the phosphorylation of D-ribose to D-ribose 5-phosphate, and GalK, responsible for the phosphorylation of α-D-galactose to α-D-galactose 1-phosphate represent the entry points for both of these sugars into the metabolism. Phosphorylation prevents them from leaking out of the cell again, due to the increased hydrophilicity (Bar-Even et al., 2012). The RK has a modest 50% increased activity with GTP compared to ATP, which is on par with what was observed previously with the XK from *P. thermosuccinogenes*, another C_5_-sugar kinase (Koendjbiharie et al., 2018). The difference is significantly higher for GalK, which has over sixfold the activity with GTP. For GK, another C_6_-sugar kinase, the difference was shown to be even bigger in *P. thermosuccinogenes*, since it could not use ATP at all.

Following these results, RK and GalK were purified via affinity chromatography, in order to determine the kinetics for ATP and GTP, as that could still vary significantly – and possibly rule out a physiologically function of either ATP or GTP for those two enzymes. Result of the assays to determine the kinetics are shown in Table 4. The affinity constants (K_M_) of RK for both ATP and GTP were found to be comparably low (i.e., high affinity); 0.028 mM and 0.154 mM, respectively. The turnover number (k_cat_) was found to be 85% higher for GTP, compared to ATP, corresponding to what was found in the assays with the non-purified enzyme. GalK has a much higher affinity for GTP compared to ATP, with K_M_ values of 3.98 mM and 0.035 mM for ATP and GTP, respectively. Furthermore, k_cat_ was found to be roughly 2.5 times higher for GTP. Intracellular concentrations of ATP and GTP are not known for *P. thermosuccinogenes*. However, In exponentially growing *E. coli*, yeast, and mammalian cells, ATP concentrations were found to be quite well conserved in the range of 2 – 10 mM. For GTP, it is in the range of 1.6–15 mM in *E. coli*, and 0.2–0.7 mM for yeast, and mammalian cells (Park et al., 2016). Considering this, it is reasonable to assume that for RK *in vivo*, both ATP and GTP are saturated, with GTP being the moderately preferred substrate – but with both ATP and GTP-dependent activity occurring. For GalK *in vivo*, GTP is certainly saturated, whereas ATP is likely not. Adding to that the big difference in turnover number, it seems that GTP is the physiological relevant cofactor.

**Table 4:** Affinity constants (K_M_) in mM and turnover numbers (k_cat_) in U/μmol of ribokinase, galactokinase, and the ATP/GTP-dependent 6-phosphofructokinase for ATP and GTP.

Enzyme	Locus tag	ATP		GTP	
		k_cat_	K_M_	k_cat_	K_M_
ribokinase	CDQ83_03295	8.11 ⋅ 10^3^	0.028	14.4 ⋅ 10^3^	0.154
galactokinase	CDQ83_02810	1.07 ⋅ 10^3^	3.98	2.56 ⋅ 10^3^	0.035
6-phosphofructokinase	CDQ83_07225	3.19 ⋅ 10^3^	0.155	3.58 ⋅ 10^3^	0.016

Additionally, the purified RK and GalK were tested with PP_i_ as a cofactor by using Malachite Green to detect released orthophosphate, but no activity was detected.

Acetate kinase represents the last step in the fermentation pathway to acetate, where acetyl phosphate is used to convert NDP to NTP, an important mechanism for fermentative organisms to produced extra ATP (equivalents). Conversely, AK can also be the first step in many microorganisms for the production of acetyl-CoA from acetate (Ingram-Smith et al., 2006). Of the 13 enzymes tested, AK was the only one where the *E. coli* background activity could not be removed with the heating step, since AK denatured as well, suggesting that it is relatively thermolabile. Nevertheless, background activity was negligible in the untreated extracts. In the direction from acetate to acetyl phosphate, the AK of *P. thermosuccinogenes* has a sevenfold higher activity with ATP compared to GTP. The acetate-forming direction is the more physiological relevant direction, since *P. thermosuccinogenes* produces acetate. However, no simple – real-time – method exists to measure the rate in the acetate-forming direction using different cofactors.

6-phosphofructokinase catalyzes the phosphorylation of β-D-fructose 6-phosphate to β-D-fructose 1,6-bisphosphate and is in many organisms one of the key regulatory steps of the glycolysis. Three orthologous 6-PFKs are present in *P. thermosuccinogenes*, that were all assayed for cofactor usage. Previous work only showed PP_i_-dependent activity in *P. thermosuccinogenes* CFE (Koendjbiharie et al., 2018). Based on expression data, and the annotation by Prokka, it seemed most likely that CDQ83_11320 is responsible for this activity (Koendjbiharie et al., 2018), as is now confirmed by the heterologous expression in *E. coli*. One of the other isoforms, CDQ83_07225, is active with both ATP and GTP at comparable levels. The measured activity is much lower than for CDQ83_11320, but the activities are normalized for CFE protein, and not for the amount of active enzyme, so the activities cannot be compared directly. The SDS-PAGE also shows that expression of CDQ83_11320 was higher compared to CDQ83_07225 (Supplementary Figure S3). For CDQ83_10650 no activity was measured at all, including for the non-heat treated extracts.

CDQ83_07225 – the 6-PFK active with both ATP and GTP – was purified via affinity chromatography, to check whether there are notable differences for the kinetics of ATP versus GTP, as was done with RK and GalK (Table 4). The K_M_ is 0.155 mM for ATP and 0.016 mM for GTP, and the turnover numbers are virtually equal for ATP and GTP, which are both most likely at saturating concentrations *in vivo*, indicating that there is not one preferred cofactor for CDQ83_07225.

Excluding the phosphotransferase system, there are three enzymatic reactions known for the direct interconversion of PEP and pyruvate: PK; PPdK; and PEP synthase (PPS; also called pyruvate, water dikinase). Since these three enzymes of the so-called “PEP-family” share a common evolutionary origin (Enriqueta Muñoz and Ponce, 2003), and since the annotations in *P. thermosuccinogenes* are ambiguous (CDQ83_03625 was annotated as PK by NCBI, as PPdK by RAST and as hypothetical by Prokka), the three orthologs/paralogs were each tested for PK, PPdK, and PPS activity, also with different cofactors (ADP/AMP vs. GDP/GMP). For CDQ83_03625, no activity was detected in any of the three assays, shown in Table 3. CDQ83_07455, annotated as PPdK, also showed PPdK activity with AMP, but not with GMP. CDQ83_09600, annotated as a PK, showed PK activity with both ADP and GDP, accordingly. ADP-dependent activity was approximately 50% higher than GDP-dependent activity.

Table 5 shows the results of the assays with the three glyceraldehyde 3-phosphate dehydrogenase orthologs (GAPDH) and the isocitrate dehydrogenase (IDH), all tested for NAD^+^ and NADP^+^-dependent activity. GAPDH is responsible for the two step reduction and phosphorylation of glyceraldehyde 3-phosphate to D-glycerate 1,3-bisphosphate. The exergonic reduction, typically with NAD^+^, drives the endergonic phosphorylation, which then enables the formation of ATP by PGK. The reverse – gluconeogenic – reaction is also catalyzed by GAPDH, but typically uses NADPH. Organisms often have both a NAD^+^ and a NADP^+^-dependent GAPDH, used in glycolysis and gluconeogenesis, respectively (Fillinger et al., 2000). Of the three GAPDHs *P. thermosuccinogenes* had annotated, only two (CDQ83_04880 and CDQ83_10590) showed any activity in the assays, which was NAD^+^-dependent in both cases. CDQ83_10590, which showed the highest activity in the assays, is the ortholog that is also expressed during growth on glucose and xylose (Koendjbiharie et al., 2018). CDQ83_07070, for which no activity was detected, belongs to the class II (archaeal) GAPDHs, for which no functional evidence in bacteria exist. Nevertheless, it does appear to be present in handful of bacterial genomes, according to the InterPro database.

**Table 5:** Enzyme assays with *E. coli* cell-free extract containing heterologously expressed GAPDH orthologs and isocitrate dehydrogenase from *P. thermosuccinogenes*, to determine the cofactor involved in the oxidation (NAD^+^ or NADP^+^).

Assay	Locus tag	Annotation	Activity	
			NAD^+^	NADP^+^
Glyceraldehyde 3-phosphate dehydrogenase	CDQ83_04880	type I glyceraldehyde-3-phosphate dehydrogenase	0.20 ± 0.01	ND^∗^
	CDQ83_07070	type II glyceraldehyde-3-phosphate dehydrogenase	ND	ND
	CDQ83_10590	type I glyceraldehyde-3-phosphate dehydrogenase	0.57 ± 0.19	ND
Isocitrate dehydrogenase	CDQ83_08625	isocitrate dehydrogenase [NADP(+)]	ND	45 ± 5

*Reaction velocities are given in μmol/mg cell-free extract protein/min ± standard deviation. ^∗^Not detected: value close to zero and not significantly different from empty vector control.*

Isocitrate dehydrogenase is responsible for the oxidative decarboxylation of isocitrate to form α-ketoglutarate and CO_2_, using NAD(P)^+^. Bacteria most commonly rely on NADP^+^-dependent IDHs, however, the ones relying on NAD^+^-dependent IDH have in common that their TCA-cycle is incomplete, as well as the absence of an isocitrate lyase (Zhu et al., 2005; Wang et al., 2012). The IDH from *P. thermosuccinogenes* was found to be dependent on NADP^+^.

## Discussion

### Sugar Kinases

It was already known that GK and XK from *P. thermosuccinogenes* prefer GTP over ATP (Koendjbiharie et al., 2018). Adding RK and GalK to that list, it becomes apparent that using GTP could be the prevailing mechanism for the phosphorylation of monosaccharides. There also appears to be a difference between C_6_ and C_5_ sugars, as GK and GalK greatly prefer GTP over ATP and XK and RK only moderately. At this point, the implications of these observations are not clear, but they might eventually help in understanding the driving factor behind the parallel use of different phosphoryl carriers in the energy metabolism – some ideas are already discussed below.

### Acetate Kinase

The AK has an apparent preference for ATP over GTP, in the acetyl phosphate forming direction. The reverse, acetate forming direction, however, is the physiological relevant direction, as acetate is one of the main fermentation products. One would easily be tempted to assume that maximum velocities with different substrates scale proportionally to each other in the reverse direction. However, emanating from the complex interactions of enzymes with their substrates, there is really no fundamental reason why the kinetics in one direction could be used to predict the kinetics in the other direction. The thermodynamics of PK restrict it from being used for detection of ATP/GTP formation in the manner it was used for ADP/GDP detection in the acetyl phosphate forming direction. ATP formation could easily be measured via an assay relying on luciferase, of course. However, luciferase is not compatible with GTP, and can therefore not be used to assess preference of ADP versus GDP. Off-line methods, that do not rely on the real-time spectrophotometric measurement are still a possibility, but such methods are exceedingly more laborious for studying kinetics and not considered for this study. An attempt was made to use the here described ATP/GTP-dependent 6-PFK for detection of both ATP and GTP production by acetate kinase in a coupled assay with 6-PFK, aldolase, triose-phosphate isomerase, and glycerol-3-phosphate dehydrogenase. However, it was found that the substrate, acetyl-phosphate, inhibited one (or more) of the coupled enzymes (data not shown), rendering the method unreliable.

The relatively low thermo-stability of AK from *P. thermosuccinogenes* was already observed earlier (Sridhar et al., 2000), as well as for the AKs from *H. thermocellum*, *Thermoanaerobacter brockii*, and *Moorella thermoacetica* (Schaupp and Ljungdahl, 1974; Lamed and Zeikus, 1980; Sridhar et al., 2000). It is fascinating that it appears to be a rather common phenomenon, and it implies there might be an evolutionary pressure for the AK to become unstable at temperatures only slightly above the organisms optimal growth temperature.

### 6-Phosphofructokinases

Three only very distantly related families of 6-PFK are known: The PFKA family; PFKB, belonging to the ribokinase family of sugar kinases; and the archaeal ADP-dependent 6-PFK family (Ronimus et al., 2001; Merino and Guixé, 2008; Cabrera et al., 2010). Best known is the PFKA family, to which most 6-PFK belong, including the three isoenzymes *P. thermosuccinogenes* possesses. The phylogeny is of PFKA is complex, due to highly prevalent lateral gene transfer and phosphoryl donor change within the PFKA family. Originally, three separate phylogenetic clades were recognized (I, II, and III) that are now expanded into seven distinct clades (B1, E, P, LONG, SHORT, X, B2, and III) (Siebers et al., 1998; Bapteste et al., 2003). Each of the three 6-PFKs from *P. thermosuccinogenes* belongs to a separate clade: The PP_i_-dependent 6-PFK (CDQ83_11320) to B2, the ATP/GTP-dependent 6-PFK (CDQ83_07225) to B1, and the 6-PFK without detected activity (CDQ83_10650) to III. CDQ83_10650 did contain the atypical Gly_117_ and Lys_137_ residues in the active site that are associated with ATP-dependent activity (Bapteste et al., 2003). *H. thermocellum* only has homologs to CDQ83_11320 and CDQ83_07225, the isoenzymes for which we were able to detect activity. It is not uncommon for bacteria to have multiple 6-PFKs. For example, *Clostridium perfringens* also has homologs from the B1, B2, and III clades (Bapteste et al., 2003), and in the methylotrophic actinomycete *Amycolatopsis methanolica*, PP_i_-6-PFK was found to be active during growth on glucose, and ATP-6-PFK during growth on C_1_ compounds (Alves et al., 2001). The fact that *P. thermosuccinogenes* has isoenzymes for the same reaction using different phosphoryl carriers does, however, again raise the question why *P. thermosuccinogenes* uses several different phosphoryl carriers for it energy metabolism in the first place.

### Phosphoenolpyruvate to Pyruvate

**FIGURE 2:**
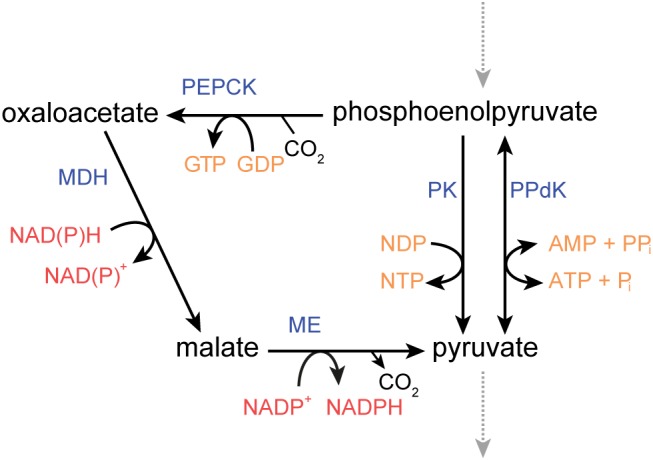
Metabolic routes connecting phosphoenolpyruvate and pyruvate in *P. thermosuccinogenes*, including the malate shunt via oxaloacetate and malate. ME, malic enzyme; MDH, malate dehydrogenase; PEPCK, phosphoenolpyruvate carboxykinase; PK, pyruvate kinase; PPdK, pyruvate, phosphate dikinase.

Three genes of *P. thermosuccinogenes* were annotated to be either PK, PPdK, or PPS. All three were tested for these three activities. It was clearly shown that CDQ83_09600 represents a PK, an enzyme well known not to be very specific toward nucleoside diphosphates (Bârzu et al., 1976; Chakrabarty, 1998), and that CDQ83_11320 represents a PPdK, functional with AMP, and not with GMP. Neither is expressed during growth on glucose or xylose at levels comparable to other glycolytic enzymes (Koendjbiharie et al., 2018). It is therefore questionable that they contribute significantly to the conversion of PEP to pyruvate. Instead, pyruvate is likely formed through the malate-shunt, comprised by PEPCK, malate dehydrogenase, and malic enzyme (see Figure 2; Taillefer et al., 2015). Nevertheless, in *H. thermocellum*, which lacks PK, it was shown that 70% of the flux from PEP to pyruvate runs via PPdK, and 30% via the malate-shunt (Olson et al., 2016). This high flux through PPdK poses another interesting question for these organisms, besides the source of all the PP_i_, namely that of AMP. Assuming that the majority of sugars taken up are converted to pyruvate, 1.4 moles of AMP need to be formed per mole of hexose consumed in *H. thermocellum*, a sizable flux that cannot be ignored. It is generally assumed that this AMP is formed via adenylate kinase (2 ADP → ATP + AMP) (Mertens, 1993; Lynd et al., 2016), but considering that at physiological relevant adenylate energy charges, the Gibbs free energy of this reaction is close to 0, it would seem to be a very inefficient use of the enzyme (Park et al., 2016), even if it was a remarkably fast enzyme, for which there is no reason to believe it is (Kerns et al., 2015). In fact, for ATP, ADP, and AMP concentrations measured in *H. thermocellum*, the net flux would favor the ADP-forming direction (Sander et al., 2017). Considering this, it appears that another mechanism must exist where AMP is formed, at a flux of comparable size to that of PP_i_.

### Pyrophosphate Usage

As explained in the introduction, the use of PP_i_ is thought to be a mechanism through which energy, or ATP-equivalents are conserved by using PP_i_, a by-product of anabolism that is otherwise hydrolysed into orthophosphate in order to maintain the low PP_i_ levels required for those anabolic reactions to proceed (Chen et al., 1990). This anabolic formation of PP_i_ is only a minor fraction of all the PP_i_ required for both 6-PFK and PPdK. Hence, there must be another mechanism by which PP_i_ is formed (Zhou et al., 2013). There is another way, however, by which the use of PP_i_ could potentially conserve energy, namely when the proton (or sodium-ion) coupling ratio is lower than that of ATPase, if the extra PP_i_ would be generated via a H^+^/Na^+^ translocating pyrophosphatase. For *Syntrophus gentianae*, it was shown that in membrane vesicles, the ratio of ATP formation to PP_i_ hydrolysis was roughly 1:3 (Schöcke and Schink, 1998). If such a coupling would exist in thermophilic *Clostridia* as well, and compared to ATP, only one-third of the protons/sodium-ions are required for the formation of PP_i_, 2/3 mole of ATP is conserved extra per dissimilated mole of glucose. Nevertheless, even a slightly more efficient coupling ratio would already signify extra energy conservation. It would almost seem obvious for such a mechanism to exist in nature, and might even explain the wide-spread occurrence of PP_i_-dependent 6-PFKs. In *Methylococcus capsulatus*, PP_i_-6-PFK and a H^+^/Na^+^ translocating pyrophosphatase were actually found to be present in the same operon (Reshetnikov et al., 2008; Khmelenina et al., 2018).

Additionally, the use of PP_i_ also allows 6-PFK as well as PPdK to operate in reverse (i.e., gluconeogenic), whereas the ATP-dependent 6-PFK and PK are strictly catabolic, as the result of the very negative Gibbs free energy for those reactions at physiological conditions (Mertens, 1991, 1993); the direct consequence of the trade-off between energy conservation and net forward flux (i.e., rate, enzyme usage, and metabolic control). This then raises the question whether the use of PP_i_ is the result of the need for extra energy conservation, or of the need for the reaction to be reversible – and the answer is likely different in different organisms. For a strictly anaerobic fermentative carbohydrate degrader such as *P. thermosuccinogenes*, reverse 6-PFK (or fructose 1,6-bisphosphatase, i.e., gluconeogenesis) activity does not seem to be an important requirement.

### GTP Usage

It was previously proposed that perhaps GTP could exist next to ATP at different guanylate and adenylate energy charges (GEC and AEC, respectively) (Koendjbiharie et al., 2018). It is generally accepted that the AEC in growing microorganisms is maintained relatively static somewhere between 0.80 and 0.95, which is absolutely crucial for cells to function (Atkinson, 1968; Chapman et al., 1971; Pradet and Raymond, 1983; Oakhill et al., 2011). Perhaps then, the GEC is allowed to be lower, or more variable. As it appears now, the main source of GTP is the PEPCK reaction, and the main drains are the sugar phosphorylation reactions discussed earlier. Disregarding anabolism, these reactions might in fact comprise a relatively closed circuit for GTP turnover. What would a lower GEC then mean for the thermodynamics for those reactions in comparison to a situation where ATP/ADP was used? The sugar phosphorylation reactions will have a lower driving force, but it is unlikely this will be impacted in any meaningful way, since these reactions generally have very negative standard Gibbs free energy, and if ATP is still used for the import of sugars via a ABC transporters, high transmembrane sugar gradients are still possible. The PEPCK, on the other hand, operates relatively close to the thermodynamic equilibrium (Zelle et al., 2010), so small changes of the product or substrate concentrations might have a relatively big impact on the fluxes. A lower GEC would lower the Gibbs free energy, or in other words, increase the ratio between the forward and reverse fluxes of the reaction, making it more efficient in terms of enzyme usage (Park et al., 2016). Furthermore, and perhaps more significant, a lower GEC would also allow the reaction to proceed at lower environmental CO_2_ concentrations, since the PEPCK reaction involves the fixation of a CO_2_ molecule. Organisms relying on PEPCK for ATP/GTP production, including natural succinate producers used in industry, are typically considered capnophilic, or “CO_2_-loving,” as they are absolutely dependent on a minimal CO_2_ concentration for growth (McKinlay et al., 2005; Song et al., 2007). Therefore, tuning the metabolism such that it would allow growth at lower CO_2_ concentrations, with a lower GEC for example, could offer a significant competitive advantage upon CO_2_ limitation. A GEC that is higher than the AEC would have the opposite effect, and assuming sugar import is indeed ATP dependent, while sugar phosphorylation is GTP-dependent, it would lower the intracellular concentrations of (non-phosphorylated) sugars. Consequently, higher rates of sugar uptake at lower extracellular sugar concentrations would be possible, potentially offering a competitive advantage under substrate-limiting conditions.

If indeed the GEC and AEC exist at different charges, which is for now purely hypothetical, it will be essential for the cell to carefully regulate the interconversion of ATP and GTP. The two main mechanisms known for balancing the degree of phosphorylation of the different NTP pools are adenylate kinase – in combination with nucleoside-diphosphate kinase – and PK (Chakrabarty, 1998). Coincidentally, *H. thermocellum* lacks a PK but possesses an adenylate kinase, where *P. thermosuccinogenes* appears to lack an adenylate kinase while possessing a functional PK, suggesting that neither is very relevant, as they seem to be lost without consequence, or that at least only having one or the other is sufficient. Another reaction that could be of importance for balancing the ATP and GTP pools is the phosphoglycerate kinase, which was shown in both thermophilic *Clostridium* species to use both ATP and GTP, but preferring ATP, while carrying a very high flux due to its role in glycolysis (Zhou et al., 2013; Koendjbiharie et al., 2018). The exact kinetics of phosphoglycerate kinase for ADP versus GDP – as well as that of other enzymes that can use both – might then be crucial to maintain the relative pools and energy charges.

The first step to investigate the hypotheses proposed here would be to actually determine whether the AEC and GEC are each maintained at different charges, for which the different nucleoside phosphate pools would need be to accurately measured during different growth conditions. A challenging endeavor, due to the labile nature of ATP and GTP – mostly the result of their high metabolic turnover, rather than actual chemical lability – leading to measured AECs/GECs that are lower than their true values (Sander et al., 2017). Perhaps, a recently developed method for real-time metabolome profiling via direct injection of living cells into a high-resolution mass spectrometer could allow the accurate determination of AEC and GEC simultaneously (Link et al., 2015).

### Glyceraldehyde 3-Phosphate Dehydrogenase

As a quintessential example of a multifunctional enzyme, GAPDH has many different functions attributed to it, aside from its catalytic role in glycolysis. For bacteria, these include functions related to virulence and DNA and RNA binding (Boradia et al., 2014; Sirover, 2014; White and Garcin, 2017). Of the three isoforms, only the two type I forms showed activity, dependent on NAD^+^, of which CDQ83_10590 belongs to the most highly expressed genes in the genome (Koendjbiharie et al., 2018). CDQ83_04880, only marginally expressed during growth on glucose or xylose might be important during other growth stages instead, such as sporulation or germination. Alternatively, any non-metabolic functions might be the primary role of CDQ83_04880 and the type II GAPDH CDQ83_07070, for which no activity was detected at all. It is uncommon for the “archaeal” type II GAPDH to be present in bacteria, and it is also not conserved among close relatives of *P. thermosuccinogenes*.

### The Enzyme Assay Method

The method used to assess cofactor usage presents a very simple and robust method to study thermotolerant and thermophilic enzymes, since no chromatography step is involved. This is owed mostly to the higher thermostability of the enzymes studied, which enables the removal of virtually all *E. coli* background activity via a simple heating step. Nevertheless, even omission of the heating step, which was required for the AK, still allowed for collection of sound data required for the identification of the enzyme’s activity and the corresponding cofactor usage, due to the impressive capacity of *E. coli* to overexpress proteins by using the T7 promoter. Of course, the omission of purification steps means that the studied enzyme can only be qualitatively characterized. The simple method used in this study can potentially be taken advantage of for a high-throughput set-up for enzyme characterization. In particular, if it were to be combined with the method published in Sévin et al. (2017) that uses mass spectrometry to identify accumulated or depleted compounds in a supplemented metabolome extract containing hundreds of biologically relevant candidate substrates.

In the case several cofactors/substrates are found to be used by an enzyme, further purification is still recommended, in order to determine the enzyme kinetics for the competing cofactors/substrates. The differences in kinetics could rule out any of the found cofactors/substrates from being physiologically relevant.

## Conclusion

To get a better insight in the parallel use of ATP, GTP, and pyrophosphate in the central energy metabolism, and for the inability to predict cofactor usage from the amino acid sequence, thirteen enzymes from *P. thermosuccinogenes* were cloned and heterologous expressed in *E. coli* to assess the cofactor usage via enzyme assays using heat-treated *E. coli* cell-free extract. The thermophilic nature of *P. thermosuccinogenes* allowed for the removal of virtual all background activity of *E. coli* from the heterologously expressed enzyme via a simple heating step, taking away the need for purification via chromatography. Three selected enzymes, ribokinase, galactokinase, and a 6-phosphofructokinase were subsequently purified further by affinity chromatography, to enable the determination of K_M_ and k_cat_ for ATP versus GTP, as they were found to use both.

As it is now clear that galactokinase and ribokinase prefer GTP over ATP, just like GK and XK, it seems that this might be the prevalent cofactor for sugar phosphorylation in *P. thermosuccinogenes*, in particularly for hexoses. There might be a more or less closed circuit for GTP-turnover between sugar phosphorylation and phosphoenolpyruvate carboxykinase, that could theoretically allow for different reaction thermodynamics then when ATP were to be used. Further research is needed to corroborate this.

Three 6-phosphofructokinases were tested. In addition to the highly expressed pyrophosphate-dependent 6-phosphofructokinase, another 6-phosphofructokinase was found to be active with both ATP and GTP. No activity was detected for the third. Both type I glyceraldehyde 3-phosphate dehydrogenases were found to be NAD^+^-dependent and for the type II “archaeal” glyceraldehyde 3-phosphate dehydrogenase no activity was detected at all. PK was confirmed to be present and to be active with both ADP and GDP, whereas another enzyme ambiguously annotated as PK did not show any activity. Pyruvate, phosphate dikinase was active with AMP, but not with GMP. Acetate kinase was found to prefer ATP over GTP in the in the acetyl phosphate-forming direction. Isocitrate dehydrogenase was active with NADP^+^.

## Author Contributions

JGK and RK conceived and designed the study. JGK and KW carried out the experiments. JGK wrote the manuscript in consultation with RK.

## Conflict of Interest Statement

The authors declare that the research was conducted in the absence of any commercial or financial relationships that could be construed as a potential conflict of interest.
