# Supplementary material for: Assessing Cofactor Usage in Pseudoclostridium thermosuccinogenes via Heterologous Expression of Central Metabolic Enzymes
Source: Front Microbiol. 2019 May 24;10:1162. doi: 10.3389/fmicb.2019.01162 (PMC6543838; doi:10.3389/fmicb.2019.01162)
Supplement: Supplementary file 1 [file Data_Sheet_1.docx]

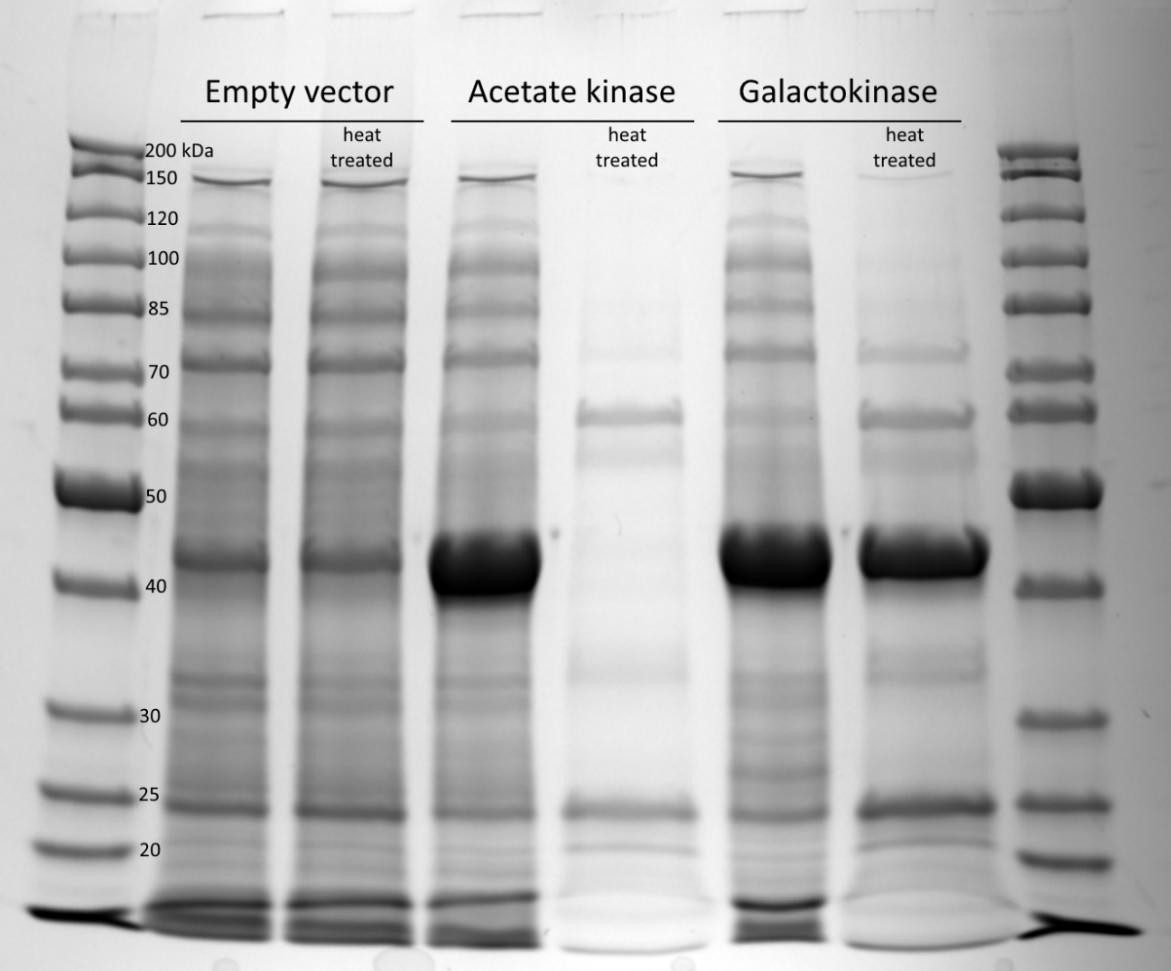

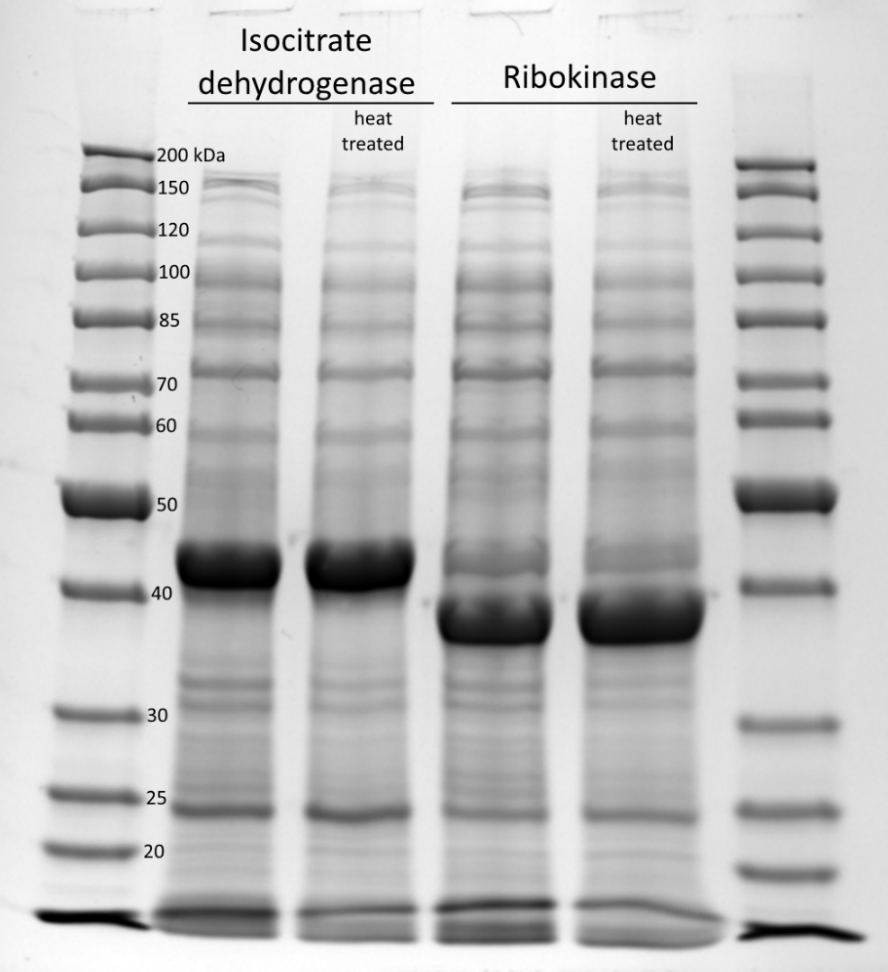


**Figure S1**: SDS-PAGE gels of (heat treated) cell-free extracts of *E. coli* expressing acetate kinase (CDQ83_07295), galactokinase (CDQ83_02810), isocitrate dehydrogenase (CDQ83_08625), and ribokinase (CDQ83_03295) from *P. thermosuccinogenes*.


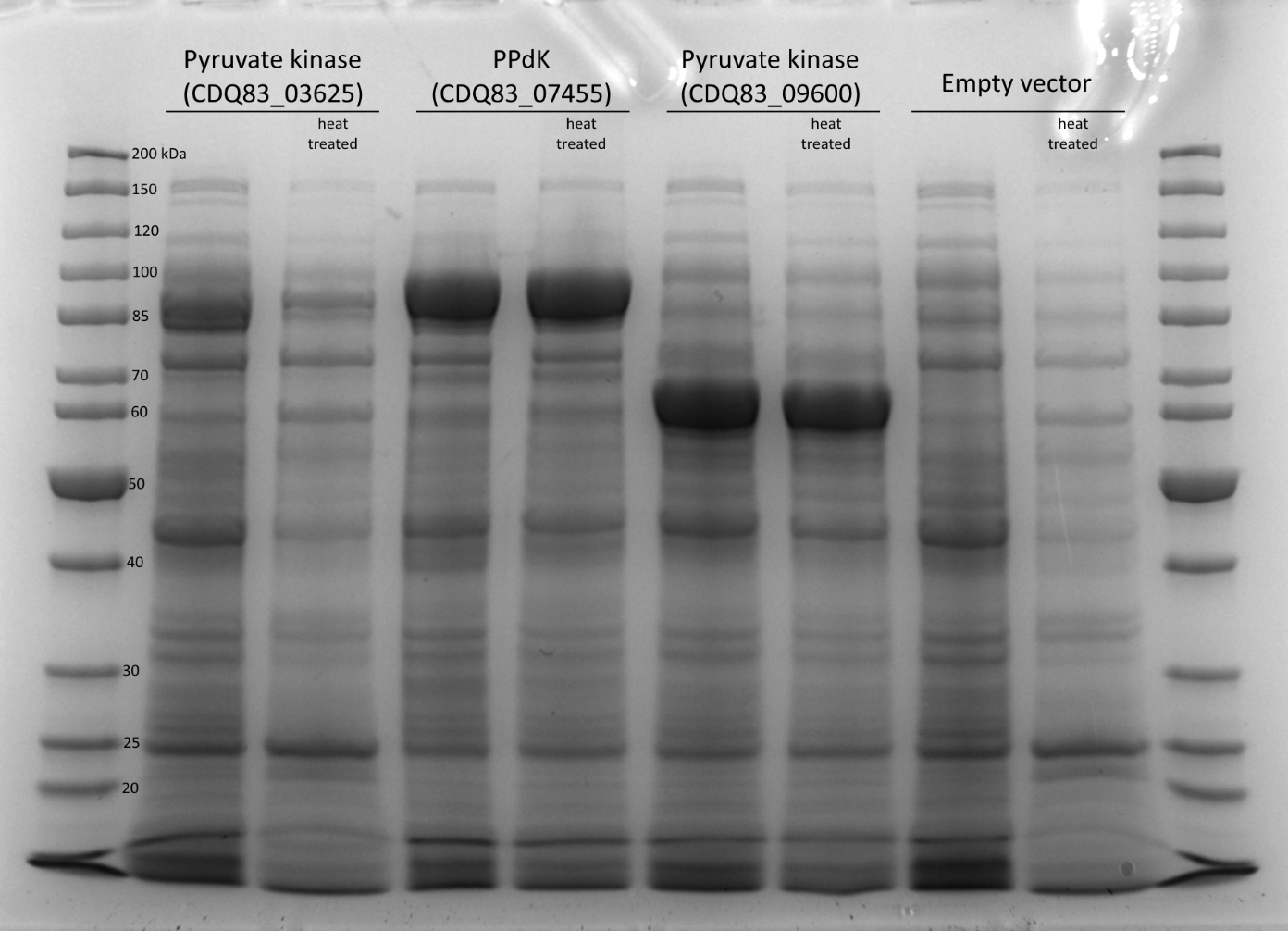


**Figure S2**: SDS-PAGE gel of (heat treated) cell-free extracts of *E. coli* expressing the three “PEP-family” enzymes (CDQ83_07295, CDQ83_02810, CDQ83_08625) from *P. thermosuccinogenes*.


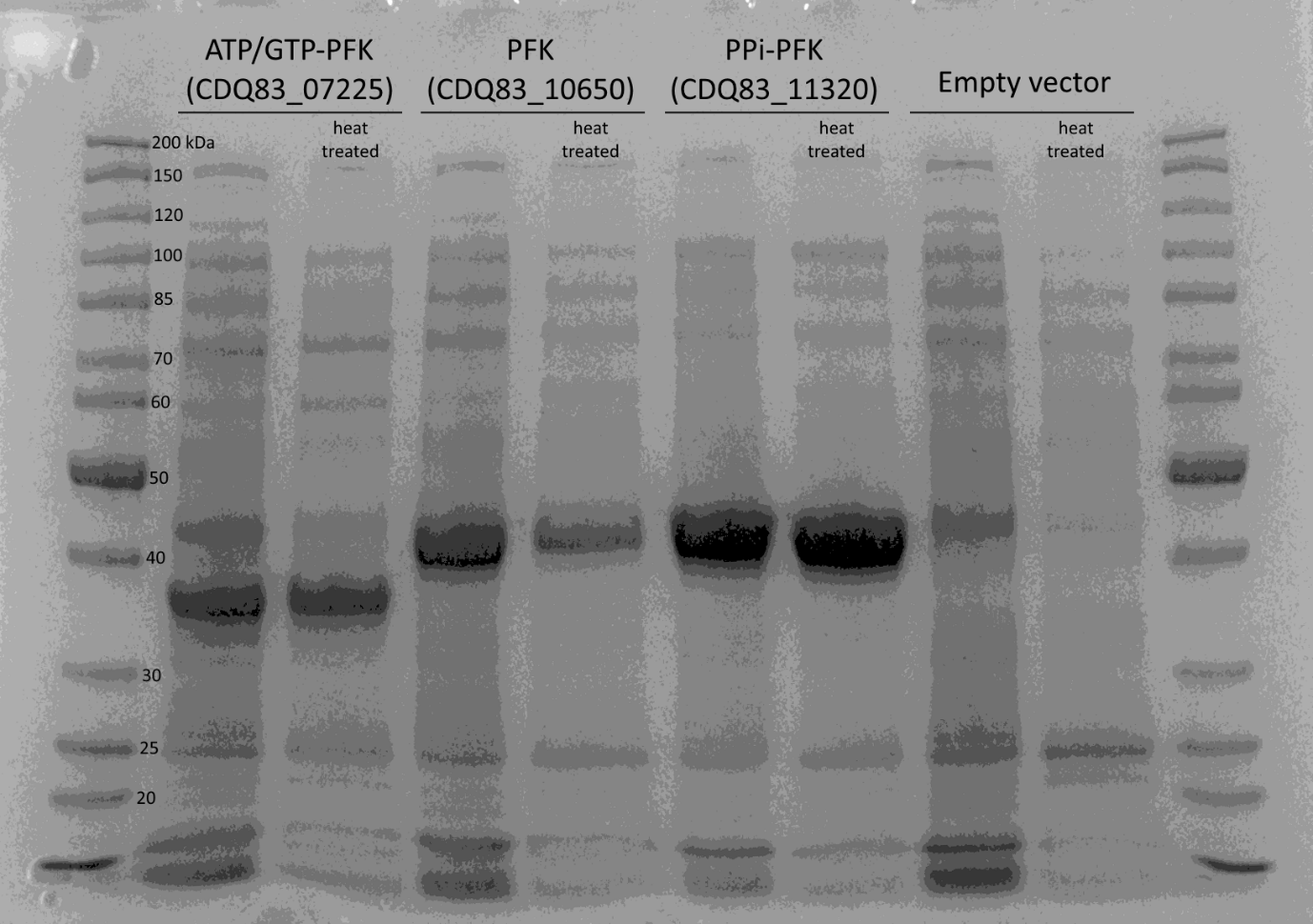

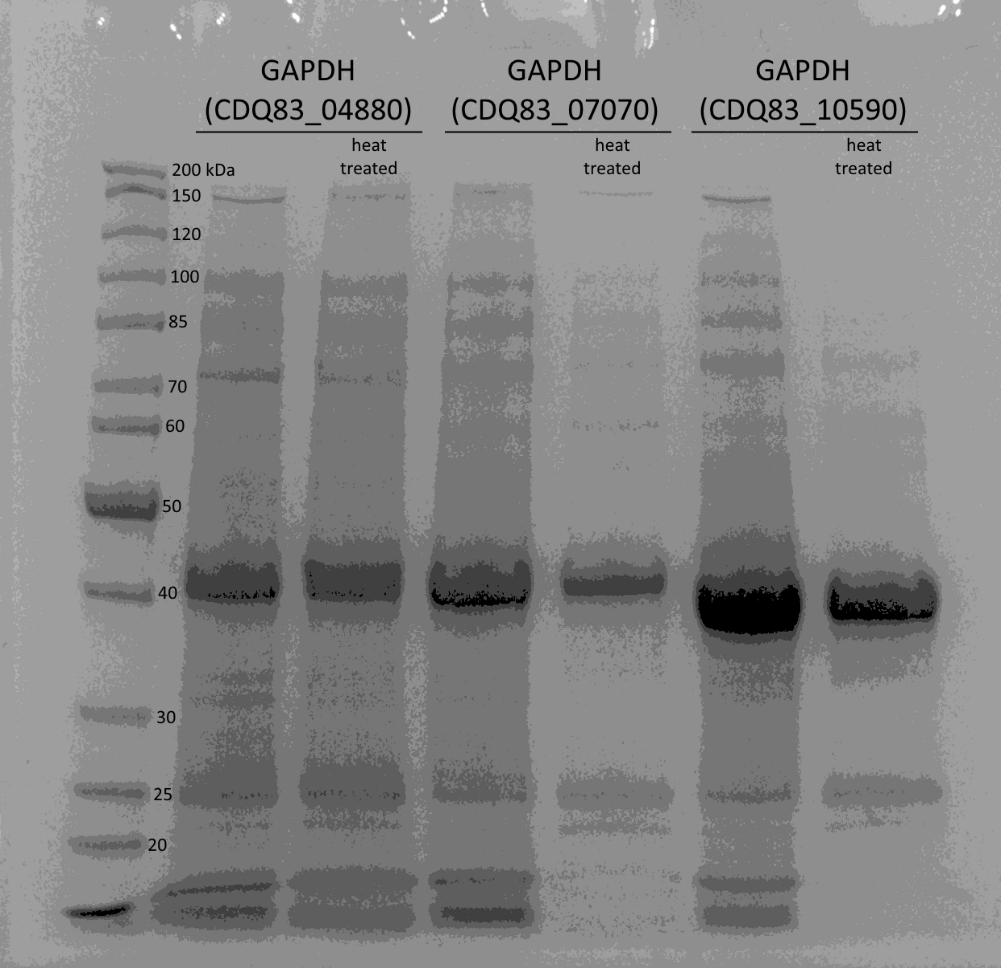


**Figure S3**: SDS-PAGE gels of (heat treated) cell-free extracts of *E. coli* expressing the three 6-phosphofructokinases (PFK; CDQ83_07225, CDQ83_10650, CDQ83_11320) and three glyceraldehyde 3-phosphate dehydrogenases (GAPDH; CDQ83_04880, CDQ83_07070, CDQ83_10590) from *P. thermosuccinogenes*.
